# Herbicide-Induced Fragmentation: Regenerative Ability of Cabomba Fragments After Exposure to Flumioxazin

**DOI:** 10.3390/biology14081023

**Published:** 2025-08-08

**Authors:** Junfeng Xu, Tobias Oliver Bickel, Steve Adkins

**Affiliations:** 1 School of Agriculture and Food Sustainability, The University of Queensland, Brisbane, QLD 4072, Australia; s4377559@uq.edu.au; 2 Invasive Plant Science, Department Agriculture and Fisheries, Brisbane, QLD 4001, Australia; tobias.bickel@dpi.qld.gov.au

**Keywords:** herbicide, invasive alien aquatic plants, cabomba, fanwort, aquatic plant management

## Abstract

*Cabomba caroliniana* A. Gray (cabomba) is an invasive alien aquatic plant (IAAP) posing a significant threat to aquatic ecosystems in Australia. Its ongoing spread is primarily driven by its rapid growth rate and ability to readily regenerate from stem fragments. Flumioxazin, an effective herbicide for controlling cabomba, has been registered for use in Australia since 2021. However, exposing cabomba to flumioxazin can induce stem fragmentation, potentially facilitating further spread. This study aims to determine whether stem fragments of cabomba following treatment at different flumioxazin doses (i.e., 25, 50, 100, or 200 ppb a.i.) can regenerate new healthy shoots that could contribute to its future spread in a new environment, in either summer or winter. This study also aims to investigate how this regrowth potential changes over time after herbicide application. Results show that flumioxazin suppressed the regeneration of replanted stem fragments in a dose-dependent manner in both winter and summer. In winter, complete regeneration was suppressed at the highest concentration tested (200 ppb a.i.), while low concentrations (25 and 50 ppb a.i.) resulted in an average 45% lower regeneration rate and 93% lower regenerated biomass than the control. In summer, suppression of regeneration was lower; at 200 ppb a.i., partial regeneration (18%) occurred with a 97% biomass reduction. At lower concentrations (25 and 50 ppb a.i.), more stem fragments regenerated (66%) and biomass reduction was lower (69%) compared to winter. Furthermore, in summer, the plants gradually regained their ability to regenerate over time after herbicide exposure, regardless of flumioxazin concentration, while no such recovery occurred in winter at any concentration. The findings show that the highest tested dose (200 ppb a.i.) can effectively suppress cabomba regenerative ability, which will greatly reduce the risk of new infestations caused by dispersed fragments, particularly in winter, when cooler temperatures and lower light are suboptimal for cabomba growth. This suggests that winter may be a more effective season for flumioxazin application. However, since some regeneration still occurred in summer, even at the highest tested dose, the highest registered label rate (400 ppb a.i.) may be necessary to ensure effective suppression under warmer conditions. Further studies are needed to evaluate this higher dose and its long-term efficacy.

## Simple Summary

Cabomba is an invasive aquatic weed that threatens freshwater ecosystems in Australia by spreading rapidly through plant fragments. While herbicide flumioxazin has been approved for cabomba control, there is a concern that it might cause plant fragmentation and regrowth elsewhere. This study investigated whether cabomba fragments can regrow after being treated with different amounts of the herbicide in both summer and winter. The research found that flumioxazin prevents the regenerative ability of cabomba in a dose-dependent manner. In winter, the highest dose completely stopped regeneration. In summer, however, some regrowth still occurred, even at the strongest tested level. Notably, fragments in summer slowly regained their ability to grow over time, but this did not happen in winter. These results suggest that applying the herbicide during winter may be more effective in stopping the weed from coming back. This research helps improve weed control strategies, reduce the spread of cabomba, and protect water ecosystems while also helping managers use herbicides more effectively and responsibly.

## 1. Introduction

*Cabomba caroliniana* A. Gray, commonly known as cabomba or fanwort, is a fully submerged invasive alien aquatic plant (IAAP) originating from the American continents [1,2]. Widely utilized as an ornamental aquarium plant, its introduction into non-native aquatic ecosystems has often resulted from improper disposal of aquarium contents [3,4,5]. Cabomba plants have a remarkable ability to form stem fragments that exhibit resistance to desiccation and can bring about the establishment of new plants in the same or new environments [5,6]. Currently, cabomba has become naturalised in over 21 countries [7], including Australia, where it causes significant socio-economic and environmental impacts, such as reducing community biodiversity, suppressing recreational activities, and increasing water body management costs. The species is rapidly spreading along Australia’s eastern coastal regions, with the most extensive infestations now found in drinking water reservoirs in subtropical southeast Queensland [1,8,9,10,11].

Various physical and mechanical methods have been employed to control cabomba [12,13,14,15,16]. However, these approaches often result in the generation of numerous stem fragments [17,18], inadvertently promoting further spread within the same water body or facilitating its spread to adjacent catchment areas through natural movement or accidental transport on machinery, thereby further exacerbating the problem [19].

Chemical control is widely regarded as an effective strategy for managing cabomba [14,15,16]. Herbicidal treatments typically produce fewer stem fragments when compared to physical or mechanical methods of control. Nevertheless, fragmentation observed in other aquatic weeds following herbicide application still raises concerns about cabomba’s further regrowth and spread after chemical control [20,21]. For instance, studies on alligator weed [*Alternanthera philoxeroides* (Mart.) Griseb.] have documented new infestations resulting from stem fragmentation after chemical control is applied [22]. Given cabomba’s ability to form and regenerate from stem fragments, it is plausible that herbicide damage could also cause stem fragmentation and that those fragments could be transported by water currents, recover over time, and form new infestations. However, existing studies have not addressed whether the herbicide-induced suppression of regenerative ability in cabomba or other aquatic weeds is permanent or temporary, which means whether cabomba could regain its regenerative ability after herbicide treatment over time. Furthermore, as a submerged aquatic plant, cabomba inhabits deeper water bodies compared to emergent plants such as alligator weed, and therefore, the stem fragments of cabomba have access to more extensive aquatic environments for dispersal, thereby posing a heightened risk of further infestation. However, there is a lack of published information on the regeneration potential of herbicide-induced stem fragments of submerged cacrophytes, including cabomba, hindering the development of longer-term effective management strategies for this weed.

Flumioxazin (2-[7-fluoro-3,4-dihydro-3-oxo-4-(2-propynyl)-2H-1,4-benzoxazin-6-yl]-4,5,6,7-tetrahydro-1H-isoindole-1,3(2H)-dione), approved for cabomba management in Australia since 2021, is an effective herbicide (Clipper^®^ Aquatic); Sumitomo Chemical Australia Pty Ltd., Level 5, 51 Rawson Street, Epping NSW, Australia) for cabomba control, with low environmental toxicity, a rapid decomposition in water, and an overall good safety profile when applied correctly [23,24,25]. Cabomba exhibits high sensitivity to flumioxazin, with research demonstrating significant biomass reductions (96%) even at low application rates of 5 ppb active ingredient (a.i.), with complete control achieved at 10 ppb a.i. [26]. These findings suggest that effective management could be achieved using doses well below the label rate of 200 to 400 ppb a.i., thereby further reducing the risk to non-target native plants and reducing the herbicide load applied to drinking water reservoirs. Nonetheless, while lower doses are effective, the risk remains that stem fragments may be formed, which later regenerate into plants, potentially increasing the risk of proliferation.

The efficacy of flumioxazin in cabomba management is known to be influenced by environmental factors such as water pH, temperature, light availability, and water currents [23,27,28]. These environmental factors vary between winter and summer and, therefore, can affect the seasonal efficacy of flumioxazin [29]. Flumioxazin is a light-dependent peroxidizing herbicide that acts by hindering heme and chlorophyll biosynthesis [30] and its half-life is highly dependent upon water pH, with rapid hydrolysis occurring in alkaline waters [25,31,32]. The seasonal variation in light intensity [29], which on average is lower in winter as compared to summer, will also affect flumioxazin efficacy, potentially allowing for greater regeneration of plants in winter than in summer. The flumioxazin label restricts its application in winter, citing that it has reduced effectiveness due to diminished light availability in winter [33]. However, given Queensland’s subtropical climate with warmer and relatively higher light intensities in the winter months, the growth of cabomba has been shown to be year-round [34], underscoring the need to understand seasonal differences in flumioxazin’s efficacy on cabomba regeneration under Queensland-specific conditions.

## 2. Materials and Methods

### 2.1. Experimental Set-Up

**Figure 1:**
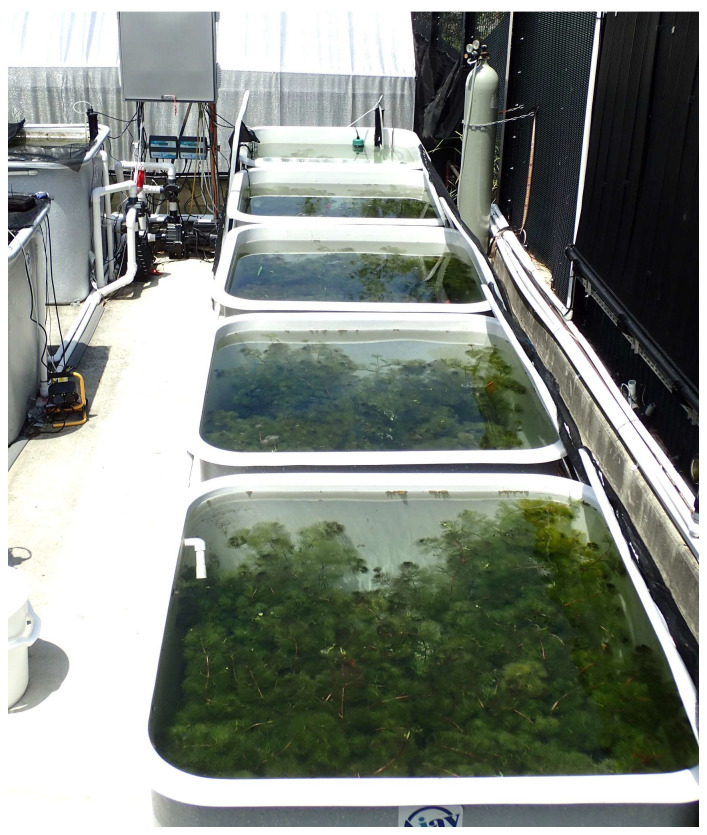
The interconnected flow-through pond system used for culturing cabomba. Five ponds were interconnected with piping to achieve an internal water circulation system using pumps and creating identical water conditions in all five ponds. A carbon dioxide (CO_2_) gas tank was connected for CO_2_ injection, visible in the background. The amount of CO_2_ injected was computerized to achieve a constant water pH of 6.5.

**Figure 2:**
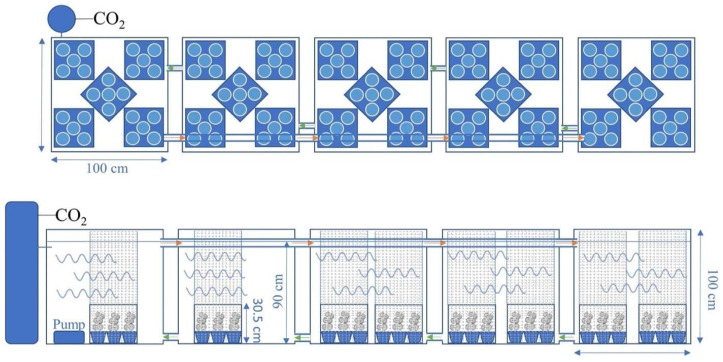
Schematic diagrams of the top and side views of the pond system used to culture cabomba before and after treatment in the regeneration experiment: Five ponds were interconnected with pipes to allow water to circulate between the ponds. The arrows indicate the direction of the circulating flow of the water. Each pond had five crates (blue squares) containing five pots with cabomba. Each crate was surrounded by a 30% generic plastic shade cloth held in place with four 100 cm bamboo stakes that reached above the water surface. The dimensions of the ponds and crates and the depth of water in the pond system are labeled. The pump that circulates water from the first to the last pond is shown in the first pond (from left to right), and a carbon dioxide injection device is connected to the first pond on the left.

The study was undertaken at the Ecosciences Precinct, Dutton Park, and carried out twice, once in winter (April to August 2022) and once in summer (December 2022 to March 2023). Each trial was conducted in two identical flow-through systems, each consisting of five 1000 L (1 m^3^) interconnected mesocosm tanks (Figure 1 and Figure 2). One flow-through system was used for the initial growth and the subsequent post-treatment growth of cabomba, while the other one was used for rinsing herbicides from plants after herbicide treatments had been applied to the plants. Both flow-through systems were filled with a culture solution suitable for supporting the growth of aquatic plants [35]. The pH in each system was electronically regulated to 6.5 by the injection of carbon dioxide (food grade, BOC, North Ryde, NSW, Australia). Liquid trace element fertilizer (Rexolin APN, Duralite, Australia) was added weekly to the flow-through systems to achieve 50 ppb a.i. of Fe to support plant development. Water quality (pH, temperature, conductivity) was monitored weekly using a YSI pro1030 pH meter (YSI Incorporated, Yellow Springs, OH, USA) throughout the trial. The water was circulated (2000 L h^−1^) within two flow-through systems to create similar conditions in each mesocosm tank, using a pump, and an ultraviolet water sterilizer in the pump (17 W, FluvalSmar, aquarium brand) was used to accelerate the breakdown of flumioxazin, potentially released from the plants after herbicide-induced injury.

### 2.2. Plant Material

Intact cabomba stems were harvested from Lake Kurwongbah (27°15′05.9″ S 152°57′36.3″ E) just prior to each trial being undertaken and immediately transported in lake water to the laboratory. Upon arrival, the stems were immediately cut into 10 cm long sections, each carrying at least three nodes with leaves and a terminal apex. Three of these stem sections, selected for their uniformity and health, were planted individually into one of 135 pots (600 mL; 125 pots with plants were used for treatment, extra 10 pots with plants were used for determining pre-treatment biomass), each filled with a substrate [circa (ca.) 840 g] consisting of washed quartz sand and peat (in a ratio of 3:1; *v*/*v*). Each pot also received 2.5 g fertilizer (Osmocote slow-release fertilizer, Scotts, Marysville, OH, USA) that had been placed in the bottom of each pot before filling, and a 1.0 cm layer of clean sand was placed on the top of each pot to reduce nutrient leaching and to retain the substrate within the pot. Five pots, each containing three stem sections, were then placed into plastic crates (for easier handling; 36.5 cm^3^), and five crates were placed into each of the five mesocosm tanks in the first flow-through system; each crate represented a single replicate. Each crate was surrounded by a 30% shade cloth (a generic brand of plastic shade cloth, Bunnings Hardware Pty Ltd., Burnley, VIC, Australia), held in place by four 100 cm long bamboo stakes that reached above the water level. The shade cloth surround was used to retain any plant fragments that might break free following herbicide application but would allow for free water movement to occur within the mesocosm (Figure 2).

### 2.3. Herbicide Treatment

**Figure 3:**
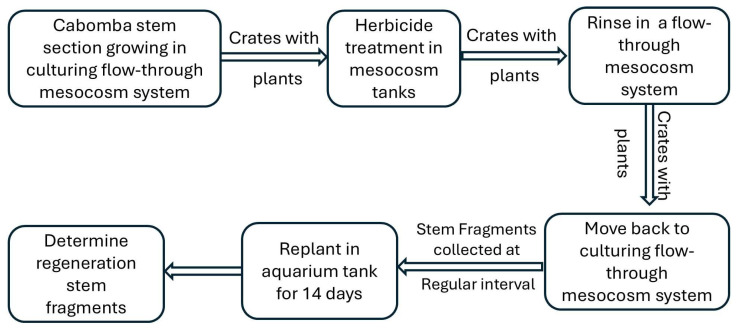
A schematic flow chart of the experimental procedure.

The stem sections within the pots were left to grow for ca. 7 (summer trial) or 12 (winter trial) weeks until they formed new plants, with multiple shoots that reached the water surface and created a dense canopy comparable to those found in the field in summer or winter, respectively (Figure 3). Pre-herbicide treatment biomass samples were collected 1 day before herbicide treatment. For this, cabomba plants biomass, excluding roots, from 10 extra pots was collected and dried at 65 °C in a laboratory oven (Thermoline Scientific dehydrating oven, Wetherill Park, NSW, Australia) for a minimum of 48 h.

Then, the five crates from each mesocosm tank in the first flow-through system were removed and treated with one of five different herbicide concentrations (i.e., 0, 25, 50, 100, or 200 ppb a.i. flumioxazin); thus, there were 5 replicate crates (each with 25 pots containing 75 original stem sections) per treatment. To apply flumioxazin, two further 800 L (1.0 × 1.0 × 0.8 m; l/w/h) mesocosm tanks were used. The appropriate amount of flumioxazin (Valor^®^ 500 WG, 500 g kg^−1^ a.i., Sumitomo Chemical Australia Pty Ltd., Level 5, 51Rawson St, Epping, NSW, Australia) for the two lowest concentration treatments (i.e., 0 and 25 ppb a.i.) were individually dissolved in 1 L of reverse osmosis contained in glass bottles and then added into the respective 800 L treatment mesocosm tanks (Figure 3). The treatment mesocosm tanks were thoroughly stirred with a paddle for 3 min to ensure the herbicide was adequately dispersed throughout the solution. The mixture was allowed to reach equilibrium before the plants were exposed to the herbicide treatments. Shade protection (100% shade cloth; a generic brand of plastic shade cloth, Bunnings Hardware Pty Ltd., Botanicca 3, Level 2 East Tower, 570 Swan Street, Burnley VIC, Australia) was provided for the top sides of the two mesocosm tanks to prevent photolysis of flumioxazin during the treatment period [27]. Exposure to the herbicide treatments was achieved by placing the five crates with plants, for the two lowest concentration treatments, directly into the individual herbicide solutions, where they remained for 2 h to ensure the efficacy of flumioxzin (minimal exposure time is 1 h) [36]. Following treatment, the crates were removed from the two herbicide solutions and allowed to drain in the open air for 5 min (enough to drain any runoff water; longer drying time would have resulted in damage to cabomba) before being rinsed in two mesocosm tanks in the second flow-through system with a pump and a UV sterilizer inside the pump, to rapidly degrade flumioxazin residues [27]. The rinsing process was undertaken for 3 min, and then, the crates were removed, allowed to drain until no further solution flowed from the plants. The crates were then placed back into the second flow-through system for a second rinse and drain (Figure 3). Then the crates were transferred back into their original positions within the initial flow-through mesocosm system (Figure 3). The remaining two higher herbicide concentrations were then applied using this same procedure, but with fresh herbicide solution in the treatment tanks and fresh rinsing solution in the mesocosm tanks.

### 2.4. Experimental Analysis

To address the first study aim, which was to determine the regenerative ability of stem sections taken from plants following herbicide application, to regrow. Cabomba stem sections were cut at random from the five lots of treated plants, now recovering in the first flow-through system. Samples were first taken when regrowth was first observed in the lowest flumioxazin treatment (25 ppb a.i.). This was 16 days after treatment in summer and 21 days after treatment in winter. Subsequent stem sections were collected every 10 days after this time. On each occasion, from treatments and controls, two 15 cm long stem sections (now and later referred to as stem fragments) were hand-cut from plants. From the herbicide treatments, the 15 cm long stem fragments were either cut from those that had fallen off the treated plants due to herbicide damage or were cut from plants, as described, for the control.

From all stem fragments, the top 10 cm portions (the portion containing the apical meristem) were cut and replanted vertically into 200 mL pots containing 1.5 g slow-release fertilizer and 280 g of the same substrate mix as described above. The pots were then placed into a 260 L aquarium tank (90 × 62 × 47 cm; l/w/h) at 25 ± 2 °C, adjusted using a thermocontrol heater and exposed to a 12/12-hour (day/night) photoperiod, simulated using two LED aquarium lights (Fluval freshwater), which provided a photosynthetic active radiation of ca. 120 µmol m^−2^ s^−1^ at the substrate level. Under these conditions, regrowth was assessed 14 days after replanting, firstly by determining the above substrate dry biomass formed in comparison to the control and secondly by determining the rate of the stem fragments to generate new shoots. Plant dry biomass was determined by drying materials at 65 °C in a laboratory oven (Thermoline Scientific dehydrating oven) for a minimum of 48 h (Figure 3).

The average water temperatures in the culturing mesocosm throughout the trials were 26 ± 2 °C in summer and 16 ± 3 °C in winter.

To address the second study aim, which was to determine the difference seen in treated plant regrowth in summer compared to winter, a simple comparison was made between the dry biomass data produced in the different treatments and the frequency of new shoot regeneration in the two seasonal trials.

### 2.5. Statistical Analysis

The treatments were randomly allocated to the ponds through a randomization process in Excel. All statistical analyses were carried out in R Studio version 4.3.2 [37]. A four-parameter exponential nonlinear model, including time after application and dose as the explanatory variables (without interaction), and using the nonlinear least squares function (nls), was fitted to the regenerated shoot dry biomass. The following formula was fitted to the data, involving four parameters (a to d) and the explanatory variables of time and dose:

(1) $$Dry\;mass=\;a-b*e^{-dose*c}+time*d$$

The three-dimensional relationship between the two instructive variables, flumioxazin dose and time after treatment, with the shoot dry biomass of the regenerated cabomba, was visualized using separate plots. One plot represents the relationship between flumioxazin dose and the shoot dry biomass of the regenerated cabomba, while the other illustrates the relationship between time elapsed since treatment and the shoot dry biomass of the regenerated cabomba. This analysis was carried out with an interaction term, which was found to be nonsignificant. Consequently, the model was simplified the model by removing this interaction term.

Dose–response models [38] were fitted with a log-logistic curve for the relationship between the application rate of flumioxazin and the mortality of cabomba fragments, with mortality defined as the inability of a stem fragment to regenerate within 14 days after replanting. The plant regeneration rate after flumioxazin treatment was calculated as the portion of treated cabomba plants recovering when compared to plant regeneration in the control. The percentage reduction in regenerated dry biomass based on herbicide dose was calculated as the proportion of regenerated cabomba plants treated with herbicide compared to the control.

## 3. Results

The pre-biomass in the winter trial was 2.3 ± 0.9 g, while it was 3.1 ± 1.1 g in the summer trial, which was ca. 35% higher than in the winter trial.

**Figure 4:**
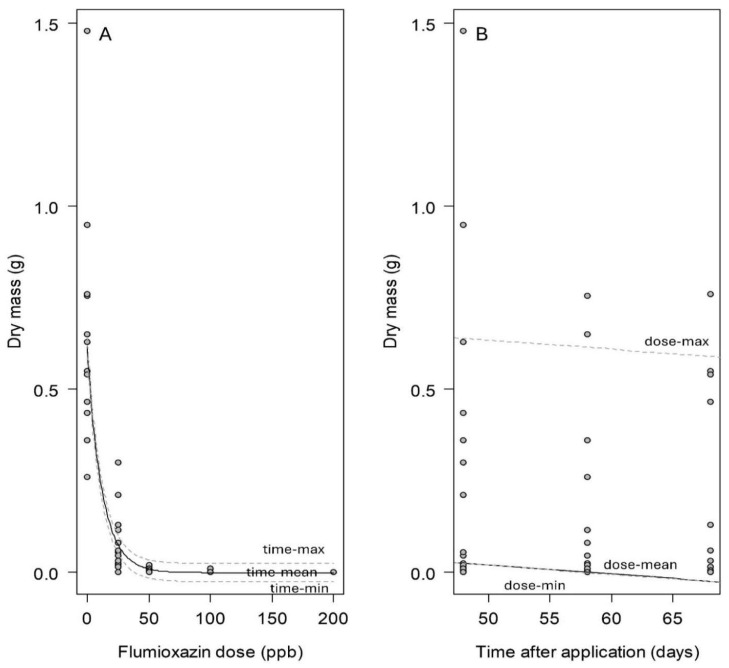
The relationship in the winter trial between the regenerated biomass 14 days after flumioxazin treatment and the flumioxazin application rate (**A**), and the time after application (**B**) (R^2^ = 0.75; AIC = −250.12). The black lines indicate the regression curve with the second parameter ((**A**): time; (**B**): dose) being at its average. The dotted lines illustrate the shift in the curve for the maximum and minimum of flumioxazin dose (**A**) and time (**B**). Parameter estimate ± SE: c = 0.083 ± 0.023 (*p* < 0.01); d = 0.002 ± 0.002 (*p* > 0.05). See Formula (1).

In winter, the recovery rate of the replanted stem fragments from cabomba following herbicide treatment ranged from 73% for the lowest dose (25 ppb a.i.; Table 1) to 0% at the highest dose used (200 ppb a.i.). The regenerated dry biomass accumulation decreased as the herbicide dose increased, showing a significant correlation (nls: df = 71, *p* < 0.001; Table 1; Figure 4A). At 100 and 200 ppb a.i., complete suppression of dry biomass production was achieved (Figure 4A).

**Figure 5:**
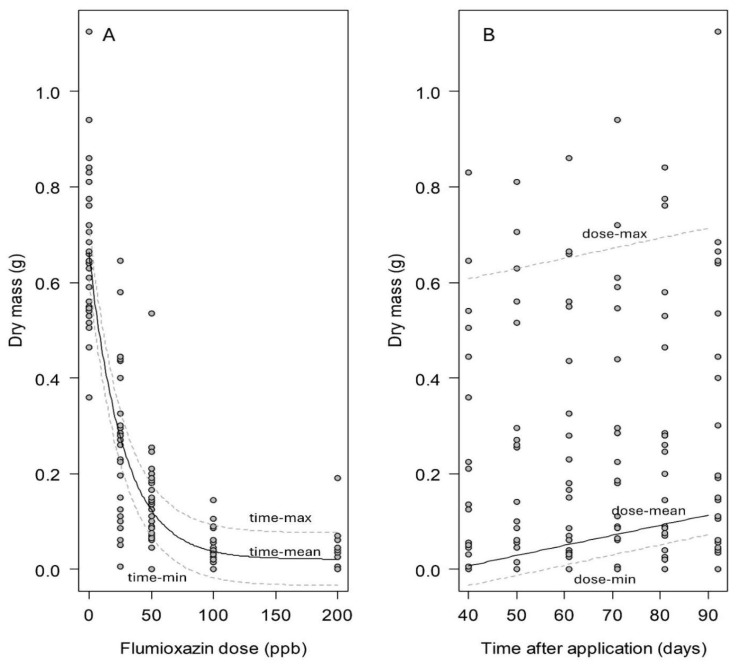
The relationship in the summer trial between the regenerated biomass 14 days after flumioxazin treatment and the flumioxazin application rate (**A**), and the time after application (**B**) (R^2^ = 0.85; AIC = −73.82). The black lines indicate the regression curve with the second parameter ((**A**): time; (**B**): dose) being at its average. The dotted lines illustrate the shift in the curve for the maximum and minimum of flumioxazin dose (**A**) and time (**B**). Parameter estimate ± SE: c = 0.036 ± 0.003 (*p* < 0.001); d = 0.002 ± 0.001 (*p* < 0.001). See Formula (1).

In summer, the recovery rate of the replanted stem fragments ranged from 72% at the lowest dose (25 ppb a.i.; Table 2) to 18% at the highest dose used (200 ppb a.i.). The regenerated dry biomass accumulation decreased as the herbicide dose increased, showing a significant correlation (nls: df = 146, *p* < 0.001; Figure 5A), but the decline in biomass was not as steep in winter. At 200 ppb a.i., regeneration was not entirely inhibited (Figure 5A).

Regenerative ability of replanted stem fragments gradually recovered over time in summer, showing a linear increase in the regenerated dry biomass (nls: df = 146, *p* < 0.001; Figure 5B). However, the time factor (estimated parameter = 0.002 ± 0.001) had a substantially smaller slope than the flumioxazin application rate (estimated parameter = 0.036 ± 0.003), indicating that the herbicide rate was the primary driver of recovery outcomes. In contrast, the winter trial showed no significant recovery over time, suggesting that the damage caused by flumioxazin was permanent in the colder conditions of winter (nls: df = 71, *p* = 0.23; Figure 4B).

**Figure 6:**
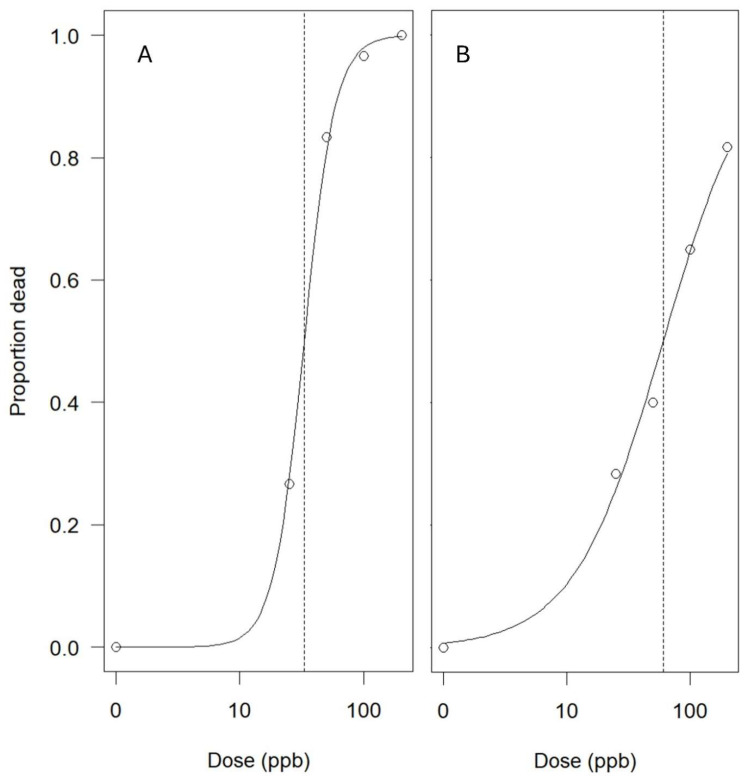
Log-logistic herbicide dose–response curves showing the proportion of dead cabomba stem fragments in the winter (**A**) and in summer (**B**). This is a measure of the stem fragments that failed to regenerate based on the flumioxazin dose. The steepest point ((**A**) EC50 = 33 ± 3 ppb a.i., confidence intervals: 27–39; (**B**) EC50 = 61 ± 7 ppb a.i., confidence intervals: 46–75; dashed line) of the curve corresponds to the dose where 50% of the cabomba stem fragments failed to regenerate.

Mortality rates in winter increased exponentially with doses from 0 to 100 ppb a.i., with 50% mortality at 33 ppb a.i. and complete mortality at 100 ppb a.i. (Figure 6A). By contrast, in summer, 50% mortality occurred at 61 ppb a.i., and complete mortality was not achieved even at 200 ppb a.i. (Figure 6B).

## 4. Discussion

Flumioxazin, applied from lower than the label-recommended up to the lowest label rate, exhibited a dose-dependent suppression on the regeneration of cabomba stem fragments. This effect was consistent across both winter and summer conditions (Figure 4 and Figure 5). However, contrary to expectations and under Queensland conditions, flumioxazin was more effective in preventing plant regeneration in winter than in summer, which is primarily reflected in two ways.

Firstly, there were higher regeneration rates under identical doses (Table 1 compared to Table 2) and a slower reduction in regrown biomass in summer than in winter (Figure 4A compared to 5A). In addition, the regeneration of stem fragments was completely suppressed under 200 ppb a.i. in winter, but not in summer. During the winter trial, the trial site was partially shaded due to the lower solar angle. Given that the efficacy of flumioxazin is affected by light intensity [30,32,39,40], it was hypothesized that summer conditions would enhance herbicide efficacy, leading to lower regeneration rates and regenerated biomass. However, the trial results suggest additional influential factors. One plausible explanation is the greater summer biomass accumulation. By comparing the pre-treatment biomass, the biomass in summer was ca. 35% higher than that generated in winter, which might create a denser canopy in summer [41,42], reducing light penetration and shielding underlying plant tissues from herbicidal effects [32]. Additionally, the increased plant biomass may have reduced the herbicide per plant biomass load, reducing its effectiveness. Another factor that may explain the lower efficacy in summer is the increased photodegradation of flumioxazin at higher UV light intensities [23,27]. While hydrolysis is a known degradation pathway [25], it was controlled in this study by maintaining water pH at a constant level, ensuring that pH effects remained uniform across seasons. Thus, due to the lower efficacy of damaging cabomba in summer, cabomba could have a stronger regeneration capacity than in winter.

Secondly, the elapsed time after flumioxazin treatment influenced cabomba regenerative ability in the summer trial but had no effect in winter (Figure 4B compared to Figure 5B). This seasonal difference in regeneration potential may be attributed to higher plant mortality seen in winter (Figure 6), resulting in a lower regeneration probability in winter. Mortality rates in winter increased exponentially with doses from 0 to 100 ppb a.i., with 50% mortality at 33 ppb a.i. and complete mortality at 100 ppb a.i. (Figure 6A). By contrast, in summer, 50% mortality occurred at 61 ppb a.i., and complete mortality was not achieved even at 200 ppb a.i. (Figure 6B). These differences suggest that plants might have suffered greater physiological damage in winter, reducing their ability to regenerate. This finding also supports our earlier hypothesis that flumioxazin is more effective in winter than in summer. Another explanation for the increased efficacy in winter could be seasonal morphological variation. Similar to other aquatic plants [43,44,45], cabomba may be structurally weaker in winter due to reduced temperature and light availability, leading to thinner stems and lower survival rates compared to summer, which develops under more favorable conditions [46,47].

Flumioxazin is considered to be one of the most efficient herbicides for controlling cabomba [23,48], which was proven through this study. When the application rate exceeded 100 ppb a.i., a concentration substantially lower than the label rate (200 to 400 ppb a.i.) of flumioxazin, over 90% of the fragments lost their regenerative capacity. Similar high efficacy has been demonstrated in other studies [26,30]; however, divergent experimental results have also been reported in some research. For example, a study conducted in Hamilton, New Zealand, used the highest label rate (400 ppb a.i.) of flumioxazin and achieved a 47% reduction in the biomass of cabomba [49]. In this study, even at the highest tested concentration of 200 ppb a.i. during the summer trial, a small number of stem fragments survived. Moreover, according to the results of this study, these surviving stem fragments may gradually regain their regenerative capacity over time. In natural environments, these surviving stem fragments are highly likely to be transported by water currents to uncontaminated areas, potentially establishing new infestations.

## 5. Conclusions

The findings demonstrate that flumioxazin reduces cabomba regrowth in both summer and winter, even at low application rates (25 ppb a.i.). Higher application rates further enhance efficacy, differing from current label recommendations [33,50], which discourage its use in cooler months of the year due to expected lower herbicide performance. The study suggests flumioxazin can be effectively used in winter in Queensland, when cooler temperatures and lower light are suboptimal for growth, potentially yielding better control than seen in summer. In practice, in winter, an application rate of 100 ppb a.i. was sufficient to prevent cabomba growth, with complete inhibition of growth achieved at 200 ppb a.i. Nonetheless, as regeneration was not entirely prevented in summer, even at the highest tested concentration, application of the maximum registered label rate (400 ppb a.i.) may be required to achieve effective suppression under warmer conditions. Further study is needed to confirm this.

This paper only looks at plant regeneration between seasons, not the control efficacy of the populations themselves. Future research should evaluate the efficacy of flumioxazin at varying concentrations for controlling cabomba and explore effective application strategies to enhance cost-efficiency while minimizing impacts on non-target plant species. Additionally, further field trials are necessary to validate the findings of this study.

## Figures and Tables

**Table 1:** The winter recovery rate of stem fragments from cabomba plants following treatment with various concentrations of flumioxazin. The data show the percentage of plants producing new viable shoots, their mean dry biomass regenerated, and the percentage reduction in the dry biomass compared to the untreated control.

Flumioxazin Dose (ppb a.i.)	Stem Fragment Regeneration Rate (%)	Regenerated Dry Biomass (mg) Plant ± SD	Biomass Reduction Relative to Control (%)
0	100	610 ± 430	
25	73	80 ± 130	87
50	17	5 ± 8	99
100	3	1 ± 5	100
200	0	0 ± 0	100

**Table 2:** The summer recovery rate of stem fragments from cabomba plants following treatment with various concentrations of flumioxazin. The data show the percentage of plants producing new viable shoots, their mean dry biomass regenerated, and the percentage reduction in the dry biomass as compared to the untreated control.

Flumioxazin Dose (ppb a.i.)	Stem Fragment Regeneration Rate (%)	Regenerated Dry Biomass (mg) Plant ± SD	Biomass Reduction Relative to Control (%)
0	100	662 ± 209	
25	72	272 ± 229	59
50	60	140 ± 153	79
100	35	41 ± 62	94
200	18	20 ± 61	97

## Data Availability

Data will be available upon request.
